# Therapeutic potential of TAS-115 in 3D breast cancer models

**DOI:** 10.1088/1758-5090/ae2400

**Published:** 2025-12-08

**Authors:** Sendegul Yildirim, Momoka Nagamine, Myoung Hwan Kim, Amanda Becceneri, Nazmiye Celik, Ilayda Namli, Todd M Umstead, Zissis C Chroneos, Gamze Tanriover, Ibrahim T Ozbolat

**Affiliations:** 1 The Huck Institutes of Life Sciences, Penn State University, University Park, PA 16802, United States of America; 2 Engineering Science and Mechanics Department, Penn State University, University Park, PA 16802, United States of America; 3 Department of Histology and Embryology, Akdeniz University, Antalya 07070, Türkiye; 4 School of Pharmaceutical Sciences of Ribeirão Preto, University of São Paulo (USP), Av. do Café, Vila Monte Alegre, Ribeirão Preto, São Paulo 14040-903, Brazil; 5 The Translational Tissue Engineering Center, Wimer Eye Institute and Department of Biomedical Engineering, Johns Hopkins University, Baltimore, MD 21231, United States of America; 6 Department of Pediatrics, Penn State University, Hershey, PA 17033, United States of America; 7 Pulmonary Immunology and Physiology Laboratory, Penn State University, Hershey, PA 17033, United States of America; 8 Biomedical Engineering Department, Penn State University, University Park, PA 16802, United States of America; 9 Materials Research Institute, Penn State University, University Park, PA 16802, United States of America; 10 Cancer Institute, Penn State University, Hershey, PA 17033, United States of America; 11 Department of Medical Oncology, Cukurova University, Adana 01330, Türkiye

**Keywords:** breast cancer, 3D models, bioprinting, heterotypic spheroids

## Abstract

Triple-negative breast cancer (TNBC) is an aggressive subtype with limited treatment options. TAS-115, a multi-receptor tyrosine kinase inhibitor, has not previously been evaluated in TNBC. Here, we investigated its therapeutic effects alone and in combination with doxorubicin (DOXO), using three-dimensional heterotypic spheroid models, including free-standing, bioprinted static, and perfused systems. TAS-115 significantly reduced cell proliferation and viability, enhanced apoptosis, and suppressed c-mesenchymal-epithelial transition/hepatocyte growth factor and PI3K/Akt/mTOR signaling. Combined treatment with DOXO further amplified these effects. In perfused bioprinted models, TAS-115 markedly inhibited tumor cell migration, highlighting its potential to limit metastatic behavior. These findings identify TAS-115 as a promising therapeutic strategy for TNBC, either as a monotherapy or in combination with chemotherapy.

## 1. Introduction

Breast cancer is the most diagnosed cancer among women worldwide and remains the leading cause of cancer-related mortality [1]. The primary determinant of mortality in breast cancer patients is not the primary tumor itself but rather metastasis. Metastasis is a multifactorial process in which the tumor microenvironment (TME) plays a crucial role. TME is comprised of various components, including the extracellular matrix (ECM), fibroblasts, adipocytes, endothelial cells, and immune cells, all of which actively contribute to key oncogenic processes, such as tumor invasion, angiogenesis, stromal genetic alterations, and immune cell activation [2]. The most critical and life-threatening stages of cancer progression are invasion and metastasis. Once a tumor acquires the ability to infiltrate surrounding tissues, it can rapidly establish secondary foci and disseminate throughout the body [3]. Once tumor cells gain the capacity to infiltrate nearby tissues, the invasion process begins. These mobile cells traverse the basement membrane and ECM, eventually reaching the intravasation stage as they enter the lymphatic or blood circulation. Metastatic cells then travel through the bloodstream, breaching the vascular basement membrane and ECM during extravasation. Eventually, they anchor in a new location, multiply, and form a secondary tumor [4]. The anchoring of cancer cells at distant organs is driven by several factors. Circulating tumor cells may become mechanically trapped in capillary peds, where they adhere through interactions between integrins or selectins and endothelial or ECM ligands such as fibronectin and collagen. Additionally, organ-specific chemokine gradients (e.g. CXCL12-CXCR4 signaling) direct site-specific homing of metastatic cells [5]. Importantly, primary tumors also secrete exosomes and soluble factors that remodel distant tissues, recruit bone marrow-derived cells, and establish an immunosuppressive microenvironment, thereby creating a pre-metastatic niche that favors colonization and outgrowth [6]. The invasion-metastasis cascade involves multiple signaling pathways, various proteins, and intricate interactions between cancer cells and stromal-derived cytokines. Each of these processes is governed by dynamic changes in gene expression, which play a pivotal role in cancer progression and metastatic potential. Focusing research on uncovering and understanding the mechanisms behind tumor cell invasion could help restrict tumor progression and, in turn, reduce mortality rates among cancer patients [4]. A key driver of these processes is mesenchymal-epithelial transition factor (c-MET), also known as hepatocyte growth factor receptor (HGFR), a well-explored pro-oncogenic protein [7] c-Met, which belongs to the MET family, is a type of receptor tyrosine kinase that is expressed on surfaces of various epithelial cells. Hepatocyte growth factor (HGF), the ligand for c-MET, functions as a potent mitogenic regulator. In cancer cells, HGF secreted either by cancer cells themselves or by surrounding stromal tissue induces the phosphorylation of c-MET. This activation, in turn, triggers multiple intracellular phosphorylations of c-MET, VEGFR, and PDGFR, thereby altering downstream signaling cascades and ultimately promoting cancer cell proliferation, invasion, and metastasis [8, 9]. Given the central role of c-MET in tumor progression and metastasis, it represents a promising therapeutic target in aggressive cancers such as triple-negative breast cancer (TNBC). This central role is largely attributed to aberrant c-MET/HGF signaling, which derives epithelial-to-mesenchymal transition, enhances cancer cell migration and invasion, stimulates angiogenesis through VEGF cross-talk, and contributes to chemoresistance, hallmarks frequently observed in TNBC.

TAS-115 is a novel multi-tyrosine kinase inhibitor that exerts its anti-tumor activity primarily through inhibition of c-MET, as well as VEGFR and PDGFR pathways. By targeting these oncogenic signaling cascades, TAS-115 disrupts multiple pro-metastatic mechanisms within the TME [10]. Preclinical studies have demonstrated the anti-tumor efficacy of TAS-115® in various malignancies, including human gastric cancer [10, 11], lung cancer [12], and synovial sarcoma [9]. However, to the best of our knowledge, there are currently no published data demonstrating the effects of TAS-115 on c-MET/HGF interactions in breast cancer and metastasis.

While clinical trials are one of the most effective methods for studying cancer cells and evaluating anti-cancer agents, ethical and safety concerns limit their widespread use. Consequently, preclinical tumor models, particularly *in vitro* culture systems, are commonly employed in cancer research. The commonly used two-dimensional (2D) monolayer cultures do not accurately recapitulate the complex TME, particularly in terms of morphology, cell–cell interactions, and cell-matrix dynamics [13]. The lack of three-dimensional (3D) structural complexity in 2D models leads to significant discrepancies in preclinical findings and reduces their translational relevance. The forces that govern cell–cell and cell–matrix dynamics in our 3D model are mediated by both biochemical and biophysical interactions. Cell–cell cohesion is primarily maintained by cadherin-mediated junctions (e.g. E-cadherin, N-cadherin), tight junction proteins, and desmosomal contacts, which generate intercellular adhesion forces and coordinate cytoskeletal tension [14]. In contrast, cell-matrix interactions are largely regulated by integrin and ECM binding (e.g. to collagen, fibronectin, and laminin), leading to the formation of focal adhesion complexes that couple the ECM to the actin cytoskeleton. These adhesive contacts transmit mechanical forces such as traction, contractility, and matrix resistance, which in turn influence migration and invasion [15]. Moreover, dynamic remodeling of the ECM by matrix metalloproteinases modifies these forces, enabling invasive cancer cell behavior [16]. Together, these physical and molecular forces provide the structural and signaling context that drives the cell–cell and cell–matrix dynamics observed in our 3D heterotypic models.

In light of these considerations, we hypothesized that inhibiting the c-MET pathway reduces cancer cell proliferation and migration by modulating downstream signaling pathways. To test this hypothesis, we investigated the effects of TAS-115, both as a monotherapy and in combination with the chemotherapeutic agent doxorubicin (DOXO). We employed 3D heterotypic TNBC spheroids incorporating both cancer cells and lung fibroblasts and the evaluated the effectiveness of TAS-115 using three different models including free-standing *in vivo* lung metastasis through heterotypic spheroids and bioprinted spheroids in static and perfused hydrogel models. This innovative approach provides a more physiologically relevant platform for evaluating the therapeutic potential of TAS-115 in breast cancer metastasis.

## 2. Materials and methods

### 2.1. Cells and reagents

Green fluorescent protein (GFP)^+^ MDA-MB-231 breast cancer cells were generously donated by Dr Danny Welch from the University of Kansas (Kansas City, KS). Normal human lung fibroblasts (NHLFs) were obtained from Lonza (Walkersville, MD). MDA-MB-231 and NHLF cells were cultured in Dulbecco’s Modified Eagle’s Medium (DMEM; Corning, New York, NY) supplemented with 10% fetal bovine serum (FBS; Life Technologies, Grand Island, NY), 1% penicillin-streptomycin (Life Technologies, Carlsbad, CA), and 1% sodium pyruvate (Corning). NHLFs were utilized between passages 3 and 8. This study did not involve human participants or primary human tissues. Therefore, ethical approval and adherence to the Declaration of Helsinki were not applicable.

Human umbilical vein endothelial cells (HUVECs) were also purchased from Lonza (Walkersville, MD) and maintained in EGM2-MV medium, prepared with a bullet kit containing 500 ml of EBM^TM^-2 Basal Medium, 25 ml of FBS, 0.2 ml of hydrocortisone, 2 ml of hFGF-B, 0.5 ml of VEGF, 0.5 ml of R3-IGF-1, 0.5 ml of ascorbic acid, 0.5 ml of hEGF, and 0.5 ml of GA-1000. HUVECs were used between passages 2–5.

All cells were incubated at 37 °C in a humidified atmosphere containing 5% CO_₂_, and the culture media were refreshed every 2–3 d. Sub-confluent cultures were detached using TrypLE Express solution (Thermo Fisher Scientific, Waltham, MA) and passaged to maintain cell proliferation.

**Visualization of NHLFs:** CellTracker Red CMTPX fluorescent dye (Invitrogen, Waltham, MA) was used to visualize NHLFs in co-culture. NHLFs were labeled with red fluorescent probes. NHLFs passaged in the previous step were allowed to reach 80% confluency. The culture medium was then removed, and a staining solution, prepared by adding 5 *µ*l of red fluorescent dye to 5 ml of DMEM, was added to flasks. Cells were incubated for 30 min at 37 °C and 5% CO_2_. Following incubation, the staining solution was removed and fresh DMEM was added. Red-stained cells were visualized using a ZEISS Axio Observer fluorescence microscope (Carl Zeiss, Jena, Germany). After imaging, cells were incubated overnight for the subsequent spheroid culture.

**Transduction of HUVECs:** HUVECs were transduced with a tdTomato lentiviral vector to enable visualization during perfusion experiments. At passage 2, HUVECs at approximately 50% confluency were transduced with an EF1 tdTomato lentiviral vector (Vectalys, Toulouse, France) at a multiplicity of infection of 20. For transduction, a mixture of the viral vector solution and complete culture medium containing 800 *μ*g ml^−1^ of polybrene (Sigma-Aldrich, St. Louis, MO) was prepared and added to culture flasks. After 8 h of incubation, the transduction mix was removed, and flasks were rinsed with 1× Dulbecco’s phosphate-buffered saline (DPBS). Fresh culture medium was added and the transduced cells were grown for 48 h. Subsequently, the brightest tdTomato-expressing cells were sorted using a MoFlo Astrios cell sorter (Beckman Coulter, Pasadena, CA) and maintained for further experiments.

### 2.2. Fabrication of tumor spheroids and determination of compaction

Following trypsinization of GFP^+^ MDA-MB-231 cells and CMTPX-labeled NHLFs, 2000 MDA-MB-231 cells and 4000 NHLFs were combined to generate heterotypic tumor spheroids. These cells were then seeded into 96-well U-bottom cell-repellent plates (Greiner Bio-One, Monroe, NC) in 100 *µ*l DMEM per well and incubated at 37 °C and 5% CO_2_ for 24, 48 or 72 h to facilitate spheroid formation. This experiment was conducted to assess the presence of CMTPX-labeled NHLFs in spheroids and evaluate the impact of cancer cells on NHLF behavior. Since dose-response assays and immunofluorescence staining were planned for subsequent experiments, non-labeled NHLFs were chosen to prevent potential interference with fluorescence-based analysis.

***α*-SMA staining:** Immunofluorescence staining for *α*-smooth muscle actin (*α*-SMA), a myofibroblast marker, was performed to evaluate the cellular architecture of spheroids. At 24 and 72 h following the 3D co-culturing of GFP^+^ MDA-MB-231 cells and NHLFs, spheroids were carefully pipetted and transferred into tubes to preserve their structural integrity. The spheroids were fixed with 4% paraformaldehyde and permeabilized with 0.1% Triton X-100 (Sigma-Aldrich) in PBS at room temperature (RT)for 15 min and subsequently blocked for 1 h at 4 °C using a solution containing 0.1% Tween-20, 10% normal goat serum (NGS), 0.3 M glycine, and 0.2% bovine serum albumin (BSA) in 1× DPBS. Following blocking, spheroids were incubated overnight at 4 °C with a mouse monoclonal anti-*α*-SMA antibody (1:100, Invitrogen) diluted in the same blocking solution. After incubation, spheroids were washed twice with DPBS and then incubated for 1 h at RT with goat anti-rabbit IgG (H + L)-Alexa Fluor 647 (1:1000, Invitrogen). Nuclei were counterstained with Hoechst 33 258 (1:200, Sigma-Aldrich) for 30 min. Finally, images were acquired using a Leica SP8 DIVE Multiphoton Microscope (Leica Microsystems, Wetzlar, Germany).

### 2.3. Drug treatment

The effective doses of Pamufetinib (TAS-115), both individually and in combination with DOXO, were determined using spheroids generated by GFP^+^ MDA-MB-231 cells and non-labelled NHLFs.

First, TAS-115 (SelleckChem, catalog no. S6764, Houston, TX) was dissolved in dimethyl sulfoxide (DMSO) (Sigma-Aldrich) to prepare a 9.64 mm stock solution (5 mg in 1 ml DMSO), which was aliquoted and stored at −30 °C. The effective dose range for TAS-115 was determined based on previous studies involving various tumor cell lines, with concentrations of 10, 100, and 500 *µ*m being selected. The stock was diluted in DMEM medium, resulting in final concentrations of 10, 100, 212, and 500 *µ*m TAS-115, corresponding to approximately 0.1%, 1%, 2%, and 5% (v/v), respectively. Similarly, DOXO hydrochloride (Tocris Biosciences, Minneapolis, MN) was dissolved in DMSO to prepare a 50 mm stock solution, aliquoted, and stored at −30 °C. The concentration range for DOXO (1.5, 6.25, and 25 *µ*m) was chosen based on optimal doses reported in studies on breast cancer cell lines [13, 17]. To account for potential solvent effects, a DMSO group was included in the experimental control, with the highest drug concentration serving as the reference. Control groups were matched to the corresponding DMSO concentration.

The untreated control group consisted of spheroids maintained in DMEM medium without drug exposure. Overall, the experimental setup was comprised of five groups: control (untreated), DMSO, TAS-115, DOXO, and TAS-115 + DOXO. TAS-115 and DOXO were applied individually or in combination, followed by incubation for 24, 48, or 72 h. At each time point, fluorescence images of spheroids were captured using the AxioObserver microscope.

### 2.4. Cell viability assay

Cell viability was assessed after 24, 48, or 72 h of the drug treatment using the Alamar Blue assay, following the manufacturer’s protocol. Briefly, spheroids were incubated with alamarBlue™ HS Cell Viability Reagent (Thermo Fisher Scientific) at 1:10 dilution in the spheroid medium for 4 h. Supernatants were then removed and the fluorescence intensity of resorufin was measured using a fluorescence-based microplate reader (Tecan, Morrisville, NC) at an excitation of 530–560 nm and an emission of 590 nm. Relative fluorescence units (RFUs) were recorded for each well, and cell viability (%) was calculated by normalizing RFUs of treated wells against those of the control group. Dose-response curves were generated using GraphPad Prism 10 (GraphPad Software, Boston, MA), and the IC_50_ (half-maximal inhibitory concentration) values, representing the concentration required to inhibit 50% of cell viability, were derived for each treatment condition.

### 2.5. Apoptosis assay

To assess caspase-3/7 activity in heterotypic spheroids treated with the IC_50_ concentrations of TAS-115 and DOXO, the Caspase-Glo® 3/7 3D Assay (Promega, Madison, WI) was performed according to the manufacturer’s instructions. Caspase-Glo 3/7 3D buffer and substrate were mixed, and 100 *µ*l of the reconstituted solution was added to spheroids that were previously exposed to TAS-115 and DOXO for 24 h. Following a 3 h incubation at RT, the solution was removed, and luminescence was measured using a microplate reader (Tecan, Morrisville, NC). Luminescence data obtained from spheroids treated with TAS-115 alone and in combination with DOXO were compared to those of untreated (control) spheroids.

### 2.6. Immunofluorescence staining of heterotypic spheroids

Heterotypic spheroids, treated with IC_50_ concentrations of TAS-115 and DOXO, were fixed with 4% paraformaldehyde in PBS at 4 °C overnight, followed by rinsing with 1× DPBS. The spheroids were then permeabilized with 0.1% Triton X-100 (Sigma-Aldrich) in PBS at RT for 15 min and subsequently blocked for 1 h at 4 °C with a blocking solution containing 0.1% Tween-20, 10% NGS, 0.3 M glycine, and 0.2% BSA in 1 × DPBS.

For immunostaining, the following primary antibodies were diluted in the same blocking solution and incubated with samples overnight at 4 °C: rat monoclonal Ki67 antibody (1:100, Invitrogen), mouse monoclonal HGF antibody (1:100), rabbit polyclonal c-MET antibody (1:100), rabbit polyclonal phospho-c-MET (Tyr1313) antibody (1:100), and rabbit polyclonal phospho-mTOR (Ser2448) antibody (1:100, Thermo Fisher Scientific). Following incubation, spheroids were washed with 1x DPBS and subsequently incubated for 2 h at RT with the following secondary antibodies: goat anti-mouse IgG (H + L)-Alexa Fluor 647 (1:1000, Abcam, Cambridge, UK), goat anti-rabbit IgG (H + L)-Alexa Fluor 568 (1:1000, Invitrogen), and goat anti-rat IgG (H + L)-Alexa Fluor 594 (1:1000, Abcam). Hoechst 33 258 (1:200, Sigma-Aldrich) was used for nuclear staining. The stained spheroids were then cleared with a solution containing 60% (v/v) glycerol (Bio Basic, Amherst, NY) and 2.5 M fructose (Sigma-Aldrich) in water at RT for 20 min before being mounted onto microscope slides. Images were acquired using a Leica SP8 DIVE multiphoton microscope (Leica Microsystems, Wetzlar, Germany) equipped with 16× water-immersion lens.

### 2.7. Cytokine analysis

Cytokine levels in culture supernatants were measured using a Luminex Discovery Assay Human Premixed Multi-Analyte Kit (R&D Systems, CA) according to the manufacturer’s instructions for supernatants. This assay was used to evaluate changes in HGFR/c-MET, TNF-*α*, IL-6, and IL-1*α*/IL-1F1 pro-inflammatory cytokines following the treatment with TAS-115 and DOXO. Heterotypic spheroids were treated at the effective time and dose, and cytokine levels were compared to those in the untreated control group.

The supernatant was collected 48 h after light irradiation for analysis. Cytokines were measured using the R&D Systems Luminex Discovery Assay Human Premixed Multi-Analyte Kit (catalog number LXSAHM) following the manufacturer’s instructions for cell culture supernatants, with untreated spheroids serving as the control. Briefly, 50 *µ*l of diluted microparticle cocktail was added to each well, followed by incubation at RT for 2 h on a shaker at 800 rpm. Then, 50 *µ*l of supernatant was added to each well. The wells were washed three times by adding 100 *µ*l of Wash Buffer. Next, 50 *µ*l of diluted Biotin-Antibody Cocktail was added, and the plate was incubated at RT for 1 h while shaking at 800 rpm, followed by another wash. 50 *µ*l of diluted Streptavidin-PE was then added and incubated for 30 min at RT while shaking. After a final wash, 100 *µ*l of Wash Buffer was added, and the plate was incubated for 2 min at RT while shaking before being analyzed within 90 min using a Luminex® analyzer.

### 2.8. Gene expression using quantitative real-time polymerase chain reaction (qRT-PCR)

To assess carcinogenic gene expression profiles by qPCR, RNA was isolated from the control group and samples treated with the effective dose of TAS-115 and DOXO for 24 h using TRIzol reagent (Life Technologies) followed by the addition of 0.2 ml chloroform per 1 ml TRIzol reagent. The mixture was then centrifuged at 12 000 × g for 15 min at 4 °C. The upper aqueous phase containing RNA was carefully transferred, and RNA was precipitated by adding 0.5 ml isopropyl alcohol per 1 ml TRIzol reagent, followed by centrifugation at 12 000 × g for 10 min at 4 °C.

The precipitated RNA was subsequently washed twice with 75% ethanol, air-dried for 10 min, and dissolved in 50 *μ*l diethyl pyrocarbonate (DEPC)-treated water and then purified using the RNA Mini Kit (Thermo Scientific) according to the manufacturer’s protocol. The concentration of RNA from each sample was determined by measuring the absorbance at a ratio of 260/280 nm using a Nanodrop (Thermo Scientific). Reverse transcription was performed using the AccuPower® CycleScript RT PreMix kit (BIONEER, Korea) according to the manufacturer’s instructions and isolated RNA was converted into cDNA. Quantitative gene expression analysis was conducted using PowerUp™ SYBR™ Green Master Mix (Thermo Scientific) on a QuantStudio 3 PCR system (Thermo Fisher Scientific).

The target genes analyzed included *c-MET, HGF, B-cell lymphoma 2 (Bcl-2), Bcl2 Associated X Apoptosis Regulator (BAX),* and *Caspase-3*. Gene sequences are provided in table 1. The fold-change in gene expression of the target genes was quantified using the 2^−ΔΔCT^ method and then normalized to glyceraldehyde 3-phosphate dehydrogenase as a housekeeping gene. The fold change of negative control (non-treated heterotypic spheroids) was set as 1-fold, and values in all groups were normalized to that of the group.

**Table 1.** Primers of the measured mRNA for qRT-PCR.

Gene	Forward and revers primers
*c-MET*	P_F_: 5′- AGT CAT AGG AAG AGG GCA TT-3′
	P_R_: 5′- CTT CAC TTC GCA GGC AGA-3′
*HGF*	P_F_: 5′- AGT ACT GTG CAA TTA AAA CAT GCG-3′
	P_R_: 5′- TTG TTT GGG ATA AGT TGC CCA-3′
*Bcl-2*	P_F_: 5′- AGC TTG GAT GGC CAC TTA C-3′
	P_R_: 5′- TGC TGC ATT GTT CCC ATA GA-3′
*Bax*	P_F_: 5′- CCC GAG AGG TCT TTT TCC GAG-3′
	P_R_: 5′- CCA GCC CAT GAT GGT TCT GAT-3′
*Caspase-3*	P_F_: 5′- TCT GTT GCC ACC TTT CGG TT-3′
	P_R_: 5′- ACT CCA CAG CAC CTG GTT ATT-3′

### 2.9. Testing of TAS-115 on a 3D bioprinted static model

To mimic ECM and better recapitulate the *in vivo* TME, a composite hydrogel formulation was prepared based on a previously published work [13]. The final hydrogel composition contained 2 mg ml^−1^ collagen (C2) and 3 mg ml^−1^ fibrin (F3). C2F3 was prepared by sequentially mixing 5 *μ*l of 10× DPBS, 0.51 *μ*l of 1N sodium hydroxide, 22.3 *μ*l EGM-2 MV medium, 50 *μ*l of 6 mg ml^−1^ fibrinogen, 22.3 *μ*l of 9 mg ml^−1^ collagen, and 1.5 *μ*l of 50 U ml^−1^ thrombin on ice.

A poly-lactic acid (PLA)-based 3D-printed structure was used as the perfusable chamber. The chamber design was created using Autodesk Inventor (Autodesk, San Rafael, CA) and fabricated using a 3D printer (Qidi Tech X-Max, QIDI Technology, China). To enhance cell adhesion, 3D-printed devices were coated overnight with a 1 mg ml^−1^ solution of poly-D-lysine hydrobromide (Sigma-Aldrich). After incubation, the devices were thoroughly rinsed with sterile deionized (DI) water to remove any excess lysine and air-dried before further use.

To evaluate tumor invasion within a biomimetic microenvironment, a total of 60 *µ*l of C2F3 was dispensed into PLA-based 3D-printed devices and allowed to partially crosslink at RT for 1 min. Tumor spheroids were individually aspirated from the fibrinogen solution using a 30G blunt nozzle under a vacuum pressure of 10–15 mmHg and bioprinted into the semi-crosslinked matrix at a proximal distance of ≈100 *µ*m from the center of device at a speed of 5 mm s^−1^ (Video S1). Following deposition, the devices were maintained at RT for 30 min to enable full crosslinking, after which an additional 60 *µ*l of C2F3 was overlaid. The device was transferred to a humidified incubator (at 37 °C and 5% CO_2_) for a complete crosslinking purpose. After 30 min, culture medium was added to the device and spheroids were incubated overnight to allow their stabilization, adaptation, and integration within the hydrogel matrix, supporting cell attachment, proliferation, and initial interactions.

To assess tumor invasion, TAS-115 was applied at its IC_50_ concentration to treatment groups. Importantly, unlike the migration analysis setup, no perfusion system was employed during this assay. Instead, TAS-115 was directly administered onto the surface of C2F3, allowing passive diffusion through the matrix and penetration into the embedded tumor spheroids to exert its therapeutic effects followed by incubation for 24, 48, or 72 h. At each time point, spheroid images were captured using a fluorescence microscope (AxioObserver). The invaded area (*µ*m^2^) was quantified using ImageJ software by subtracting the original spheroid area from the total invaded area. The degree of invasion (*µ*m^2^) was calculated based on this difference.

### 2.10. Testing of TAS-115 on a 3D bioprinted perfusable model

To investigate the migratory behavior of heterotypic tumor spheroids within the C2F3 matrix under dynamic conditions, NHLFs were suspended at a concentration of 0.5 × 10^6^ cells ml^−1^. Spheroids were bioprinted using aspiration-assisted bioprinting into a pre-filled PLA-based 3D-printed device that contained a perfusable central chamber, as previously described [13] A 30G blunt nozzle (inner diameter: 150 *µ*m) with an aspiration pressure of ∼10–15 mmHg and a bioprinting speed of 10 mm s^−1^ was utilized. A total of six spheroids were bioprinted within 3 min, positioned at proximal (∼100 *µ*m) and distal (∼500 *µ*m) distances from a stainless-steel wire that temporarily defined the vascular channel (Video S1).

Following bioprinting, constructs were incubated at 37 °C with 5% CO_2_ to allow complete hydrogel crosslinking. The stainless-steel wire was then carefully removed, creating an open microchannel, which was flushed multiple times with medium to ensure clearance. HUVECs were seeded into the channel at a concentration of 25 × 10^6^ cells ml^−1^ to establish a vessel-like lumen. To promote uniform cell attachment, the device was rotated every 30 min over a 1 h period. Constructs were subsequently in EGM-2MV medium and incubated overnight under standard conditions (37 °C, 5% CO_2_).

Unlike the static model, this model incorporated a dynamic perfusion system to mimic physiological flow. The device was connected to an external perfusion system (Reglo Ismatec, MasterFlex, Radnor, PA) and perfused with EGM-2MV medium at a constant rate of 0.7 *μ*l min^−1^ for 24 h [13]. Drug treatment was initiated by perfusing the constructs with EGM-2MV medium supplemented with TAS-115 (212.5 *µ*m) and DOXO (1.5 *µ*m), as determined in section 3.3. After 24 h of treatment under flow, perfusion was stopped, and the constructs were fixed with 1 ml of 4% paraformaldehyde overnight at 4 °C. After fixation, spheroids were washed with 1 ml of 1× DPBS and imaged using the Leica SP8 DIVE multiphoton microscope equipped with 16× immersion lens. Z-stack images were collected from defined regions across the construct, and full-volume 3D reconstructions were generated by mosaic stitching.

### 2.11. Statistical analysis

All data were presented as the mean ± standard deviation and analyzed by GraphPad 10.0.3 using one-way analysis of variance followed by the Posthoc Tukey’s multiple comparison test. ImageJ data, calculated separately for each group, were based on three independent experiments, each with four replicates. When comparing multiple groups with a single control group, a Dunnett Multiple Comparisons test was used. Statistical differences were considered significant at ^*^p <0.05, ^**^p <0.01, ^***^p <0.001, and ^****^p <0.0001. Statistical comparisons were performed against both control and DMSO groups.

## 3. Results

### 3.1. Establishment of the 3D heterotypic tumor model and determination of the individual effect of TAS-115 and DOXO

**Figure 1.**
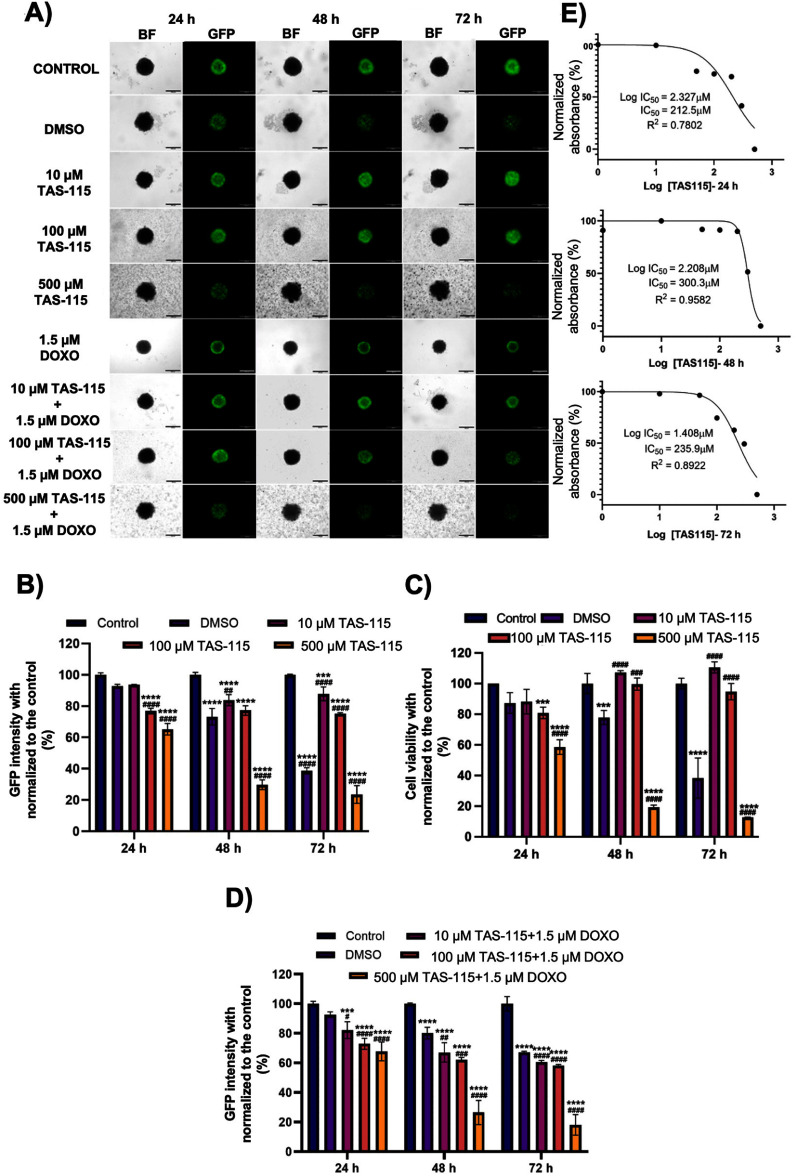
(A) Bright-field (BF) and fluorescence images of GFP^+^ MDA-MB-231 spheroids following the treatment with different doses of TAS-115 after 24, 48 and 72 h. Scale bar represents 200 *µ*m. (B) Graphical representation of normalized GFP intensity and (C) cell viability after 24 h, 48 h, and 72 h of the TAS-115 treatment. (D) Statistical analysis graphs of GFP intensity normalized with respect to the control group in spheroid images taken after 24, 48 and 72 h of TAS-115 administration (at different concentrations) to heterotypic spheroids. (E) IC_50_ values for 24 h, 48 h, and 72 h TAS-115 treatment (*n = 12*) (p^*^ < 0.05, p^**^ < 0.01, p^***^ < 0.001, p^****^ < 0.0001; compared to the control, *p*^#^ < 0.05, *p^##^*< 0.01, *p^###^* < 0.001, *p^####^* < 0.0001; compared to DMSO).

In 3D spheroid cultivation, GFP^+^ MDA-MB 231 cells appeared slightly larger at 72 h compared to 24 h, suggestive of increased proliferation, although this may also reflect reduced compactness (figure S1A). In contrast, CMTPX-labeled NHLFs showed a reduction in number and loss of their spherical morphology relative to the initial time point. This observation is consistent with previous findings from metastatic breast cancer models in which cancer cells negatively affect lung tissue [18]. In heterotypic spheroids, lung fibroblasts exhibited similar deleterious changes, including reduced numbers and altered morphology. Following morphological evaluation, *α*-SMA staining revealed intense expression within heterotypic spheroids, indicating the formation of a well-organized spheroid structure. Myofibroblasts, the key components of connective tissues, contributed to spheroid compaction (figure S1(B)). After treatment with different doses of TAS-115 and DOXO (figure 1(A)) for 24, 48, and 72 h, a dose-dependent reduction in the number of GFP^+^ MDA-MB-231 cells was observed, with the decrease progressing over time. Spheroid compaction remained intact initially but began to deteriorate at higher TAS-115 doses (500 *µ*m). To assess whether reduced viability was attributable to DMSO rather than TAS-115, experimental groups were compared to both untreated and DMSO controls. This analysis confirmed that the observed effects were drug-specific and not solvent-related.

GFP intensity significantly decreased in the 100 *µ*m and 500 *µ*m TAS-115 groups compared to the control group (*p* < 0.0001), with the most substantial reduction observed at 72 h in all TAS-115 treated groups (figure 1(B)). Normalized with respect to the untreated group, intensity decreased to ∼87% in the 10 *µ*m TAS-115- treated group, ∼75% in the 100 *µ*m group and ∼24% in the highest dose (500 *µ*m) TAS-115- treated group. Compared to the untreated group, viability decreased to ∼81% after 24 h administration of 100 *µ*m TAS-115 and to ∼59% after 500 *µ*m TAS-115 administration. In the 500 *µ*m TAS-115 group, viability decreased to ∼20% after 48 h and ∼13% after 72 h compared to the control (figure 1(C)). These results led to continuations of experiments with the lowest dose of DOXO (1.5 *µ*m) considering the individual effect of TAS-115. Furthermore, analysis of GFP intensity in spheroid images taken after 24, 48, and 72 h of incubation following co-administration of TAS-115 and DOXO revealed significant reductions in GFP intensity in the 10 *µ*m TAS-115 + 1.5 *µ*m DOXO (*p* < 0.001), 100 *µ*m TAS-115 + 1.5 *µ*m DOXO, and 500 *µ*m TAS-115 + 1.5 *µ*m DOXO groups (*p* < 0.0001) (figure 1(D)). After 48 and 72 h, GFP intensity significantly decreased in all treated groups compared to the control (*p* < 0.0001), where the intensity, normalized with respect to the untreated group, declined to ∼27% in the TAS-115 + DOXO- treated group (figure 1(D)). Notably, in the combinatorial treatment setting, GFP intensity values in the DMSO control appeared higher than those in the TAS-115-only experiments at 72 h. However, all statistical analyses were consistently performed against both untreated and DMSO controls, confirming that the observed TAS-115 effects were not attributable to solvent influence. In line with all the findings, the effective value (IC_50_) of TAS-115 was determined to be 212.5 *µ*m for 24 h of incubation, 300.3 *µ*m for 48 h, and 282.1 *µ*m for 72 h (figure 1(E)). Based on these findings, the effective concentration and incubation period selected for further experiments to evaluate the effects of TAS-115 alone and in combination with DOXO for 24 h was 212.5 *µ*m.

### 3.2. Evaluation of survival responses following the individual and combined administration of TAS-115 with DOXO in heterotypic tumor spheroids

**Figure 2.**
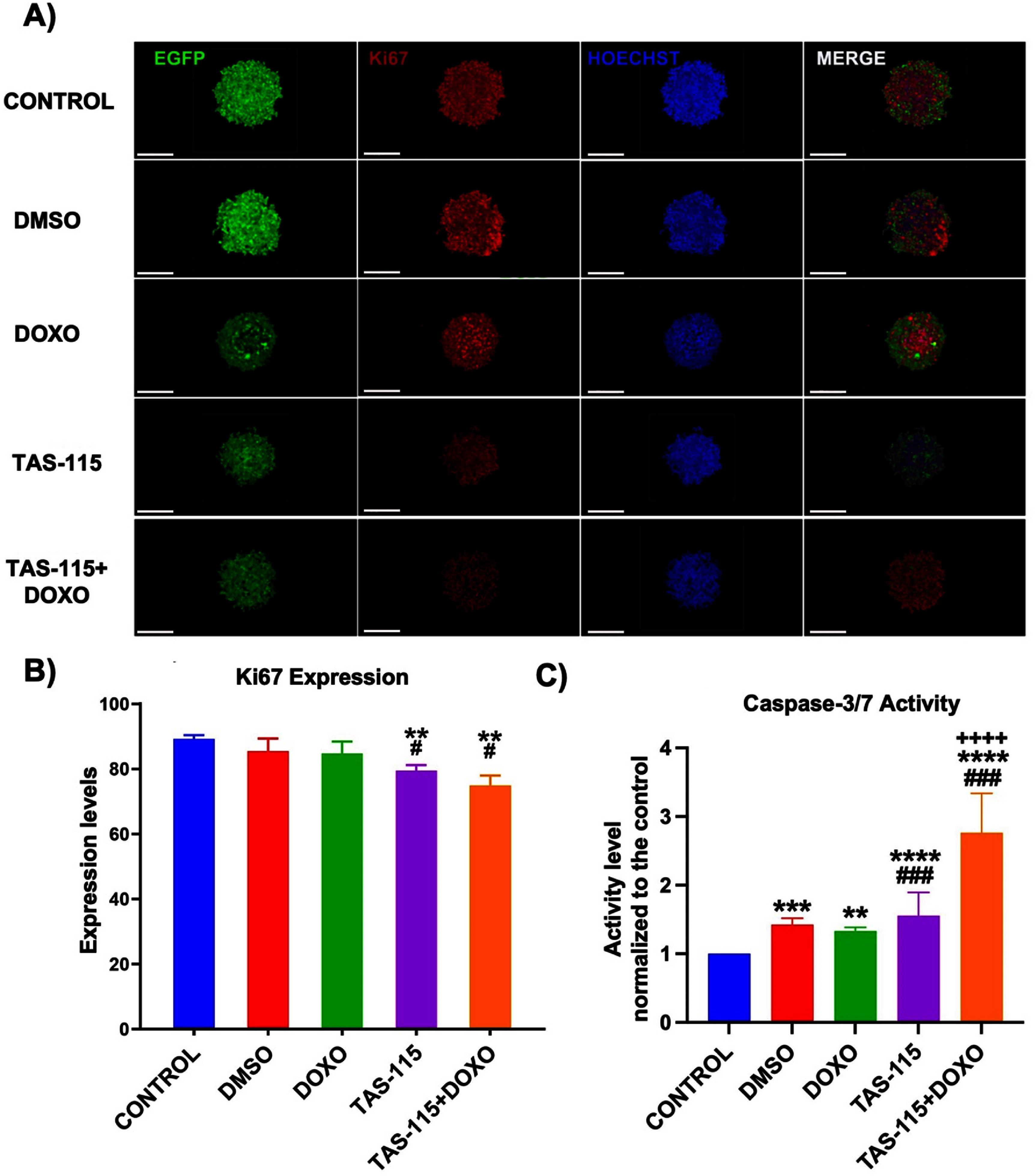
(A) Immunofluorescence images of heterotypic tumor spheroids after TAS-115 and DOXO treatment stained for Ki67 and Hoechst. Scale bar represents 200 *µ*m. (B) Graphical representation of Ki67 expression and (C) apoptosis of 24 h individual and combined treatment of TAS-115 with DOXO by the Caspase-3/7 assay normalized with respect to the non-treated control group (mean ± SD, *n*= 12) (p^*^ < 0.05, p^**^ < 0.01, p^***^ < 0.001, p^****^< 0.0001; compared to the control, *p*^#^ < 0.05, *p^##^*< 0.01, *p^###^* < 0.001, *p^####^* < 0.0001; compared to DMSO).

In this study, Ki67 staining was used to evaluate the effect of the inhibitor on tumor cells. Cells expressing Ki67 were highly abundant in the control group, whereas Ki67 intensity was notably reduced in TAS-115 and TAS-115 + DOXO groups (figure 2(A)). Quantitative analysis revealed that Ki67 expression was decreased in DMSO and DOXO groups; however, this decrease was not statistically significant (*p* > 0.05). In contrast, Ki67 expression was significantly decreased in the TAS-115 group to 80% and in the TAS-115 + DOXO group to ∼75% compared to ∼90% in the control group. Furthermore, Ki67 expression was significantly lower in TAS-115-treated (*p* < 0.05) and TAS-115 + DOX (*p* < 0.0001) groups compared to the DMSO group, suggesting that the observed inhibitory and chemotherapeutic effects were not induced by DMSO but rather due to the specific action of the compounds (figure 2(B)).

To assess whether TAS-115 induces apoptosis in heterotypic spheroids and to determine whether its combination with DOXO enhances the apoptotic activity, the Caspase-Glo® 3/7 3D Assay was performed. Caspase-3 and −7, key executors of apoptosis, were significantly activated in response to the TAS-115 and DOXO treatment, either individually or in combination. The observed increase in luminescence signals confirms the cleavage of the luminogenic Caspase-3/7 substrate, indicating elevated apoptotic activity. Compared to the control group, cell death increased by 1.42-fold in DMSO (*p* < 0.001), 1.33-fold in DOXO (*p* < 0.01), 1.89-fold in TAS-115 (*p* < 0.0001) and 3.34-fold in the TAS-115 + DOXO group (*p* < 0.0001). Additionally, the apoptotic activity was significantly higher in the TAS-115 (*p* < 0.0001) and TAS-115 + DOXO (*p* < 0.0001) groups compared to the DMSO group. When comparing the apoptotic effects of TAS-115 individually or in combination with DOXO, caspase-3/7 activation was significantly higher in the TAS-115 + DOXO group than in the TAS-115 group (*p* < 0.0001). Notably, the TAS-115 + DOXO combination resulted in the highest Caspase-3/7 activation, indicating enhanced apoptotic activity, which may reflect complementary actions of the two agents compared to TAS-115 or DOXO alone (figure 2(C)).

### 3.3. C-MET/HGF-mediated mTOR signaling following individual and combined administration of TAS-115 and DOXO in heterotypic tumor spheroids

**Figure 3.**
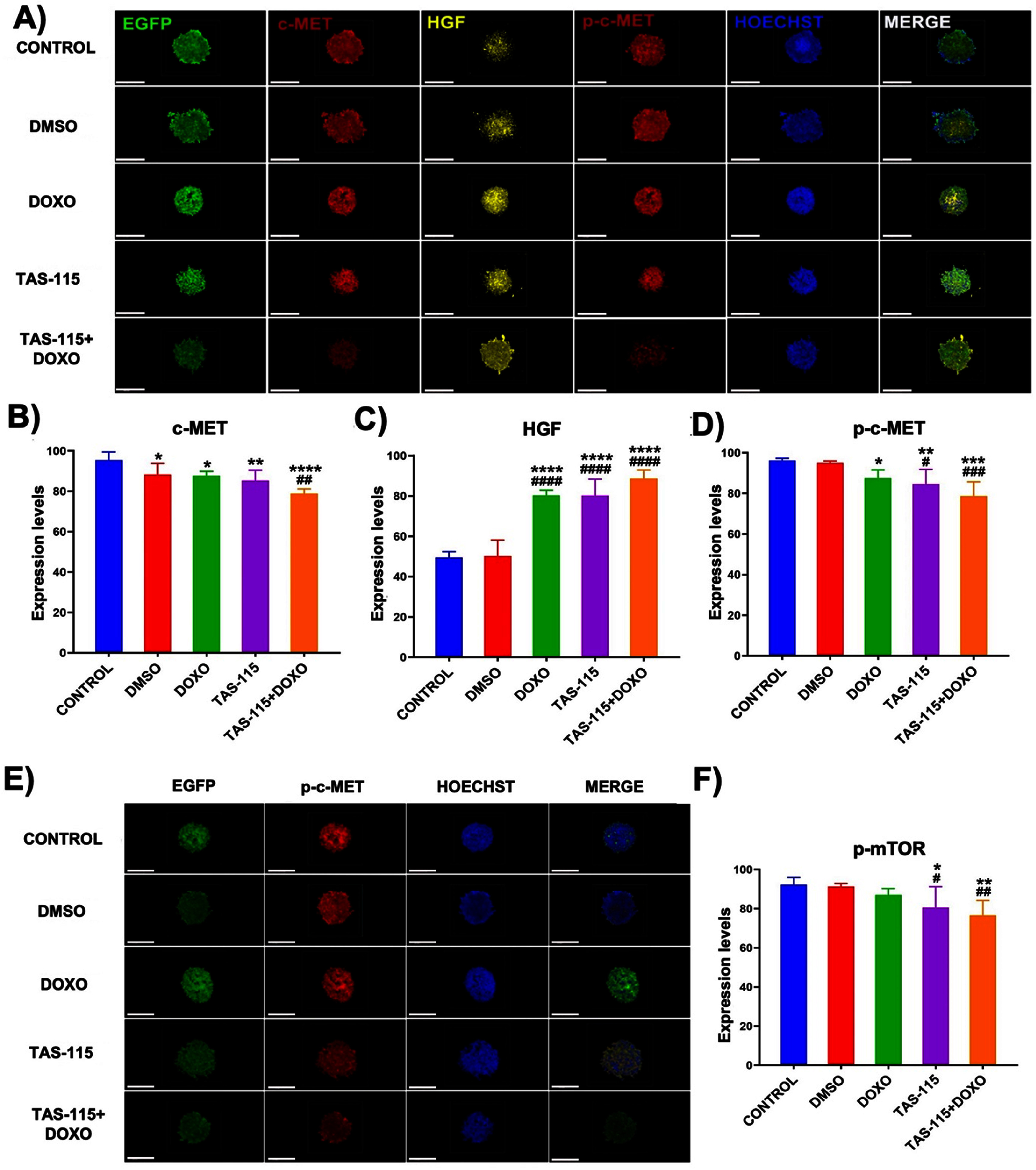
Immunofluorescence images of heterotypic tumor spheroids after the TAS-115 and DOXO treatment: (A) c-MET, p–c-MET, HGF and Hoechst expression. Scale bar represents 200 *µ*m. (B) Graphical representation of c-MET, (C) HGF, and (D) p–c-MET expressions normalized with respect to the non-treated control group (mean ± SD, *n*= 12). (E) Immunofluorescence images of heterotypic tumor spheroids after the TAS-115 and DOXO treatment showing the expression of p-mTOR and Hoechst. Scale bar represents 200 *µ*m. (F) Graphical representation of p-mTOR expression normalized with respect to the non-treated control group (mean ± SD, *n* = 12) (p^*^ < 0.05, p^**^ < 0.01, p^***^ < 0.001, p^****^ < 0.0001; compared to control, *p*^#^ < 0.05, *p^# #^*< 0.01, *p^###^* < 0.001, *p^####^* < 0.0001; compared to DMSO).

Immunofluorescence analysis was conducted to assess the effects of TAS-115, alone or in combination with DOXO, on the c-MET/HGF protein expression in heterotypic tumor spheroids. The results showed a decrease in the c-MET expression, which was mainly observed in regions containing GFP^+^ cancer cells, and a concurrent increase in HGF expression compared to the control group (figure 3(A)). Quantitative analysis showed that the c-MET expression was significantly reduced by 0.9-fold (*p*< 0.05) in DMSO and DOXO, 0.89-fold (*p* < 0.01) in TAS-115, and 0.82-fold (*p* < 0.0001) in TAS-115 + DOXO groups compared to the control. Compared to the DMSO group, the c-MET expression was lower in the TAS-115 and TAS-115 + DOXO groups, with a statistically significant decrease in the latter (*p* < 0.01) (figure 3(B)). Additionally, the HGF expression was significantly elevated in the treatment groups compared to both the control (*p* < 0.0001) and DMSO (*p* < 0.0001) groups, likely associated with increased HGF expression, which may reflect improved NHLF viability (figure 3(C)).

**Figure 4.**
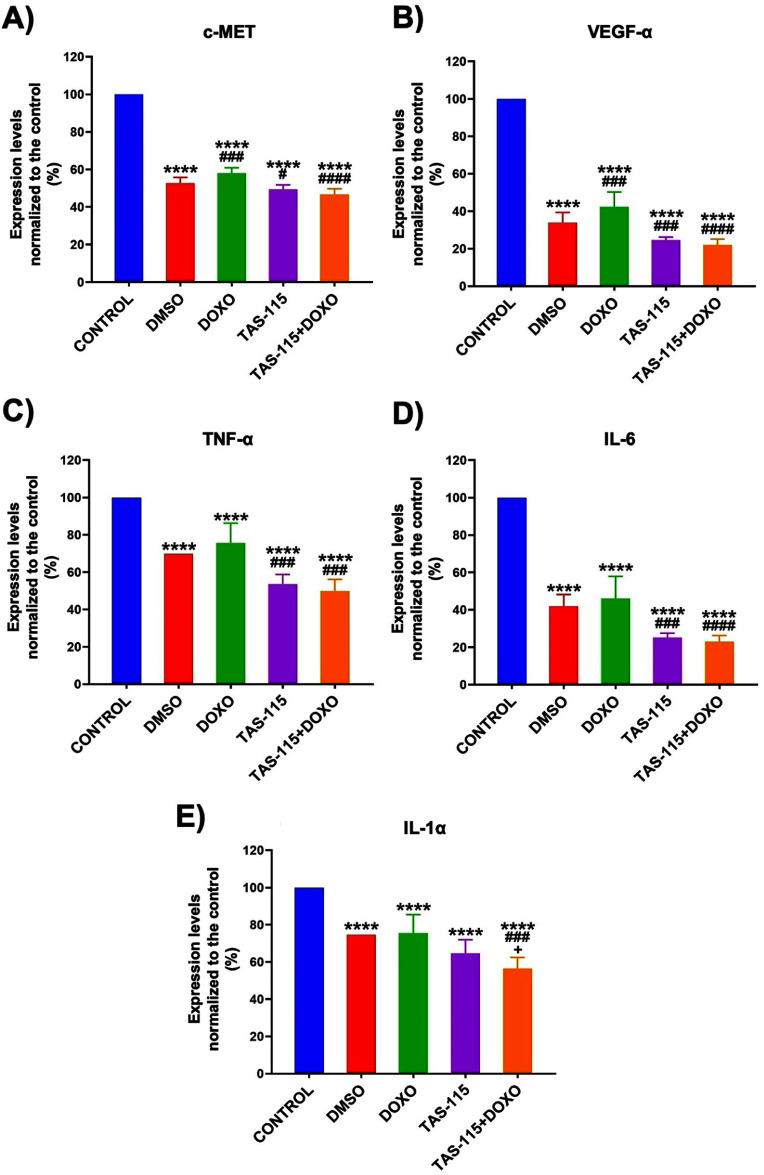
Expression levels of (A) c-MET, (B) VEGF-*α*, (C) TNF-a, (D) IL-6 and (E) IL-1α in heterotypic tumor spheroids following the TAS-115 treatment, both individually or in combination with DOXO, as assessed by Human Luminex® analysis (mean ± SD, *n*= 12) (p^*^ < 0.05, p^**^ < 0.01, p^***^ < 0.001, p^****^ < 0.0001; compared to control, *p*^#^ < 0.05, *p^##^*< 0.01, *p^###^* < 0.001, *p^####^* < 0.0001; compared to DMSO).

It is known that HGF binding to c-MET induces its autophosphorylation, leading to increased p–c-MET expression [19]. Immunofluorescence analysis revealed a parallel decrease in -c-MET and c-MET expression in the treatment groups compared to the control group (figure 3(A)). Statistical analysis confirmed a significant reduction in p–c-MET expression in the DOXO (*p*< 0.05), TAS-115 (*p* < 0.01), and TAS-115 + DOXO (*p* < 0.001) groups. This expression change was highest in the TAS-115 + DOXO group with a 0.8-fold decrease (figure 3(D)). Compared to the DMSO group, this reduction was significant in the TAS-115 (*p* < 0.05) and TAS-115 + DOXO (*p* < 0.001) groups (figure 3(D)). Phosphorylated mTOR expression, activated by p–c-MET, was the highest in the control group and decreased in DOXO, TAS-115, and TAS-115 + DOXO groups (figure 3(E)). Statistical analysis showed a significant reduction in phosphorylated mTOR levels by 0.87-fold in TAS-115 and 0.82-fold in TAS-115 + DOXO compared to control and DMSO groups (*p* < 0.0001). In addition, p-mTOR expression was lower in the TAS-115 + DOXO group compared to the TAS-115 group (*p* < 0.05), suggesting an enhanced inhibitory effect with the combinatorial treatment (figure 3(F)). In addition, the role of TAS-115 and DOXO treatment on HGFR/c-MET, VEGF-*α* and IL-6, TNF-*α*, IL-1*α*/IL-1F1 cytokine levels was also assessed. A significant reduction in HGFR/c-MET levels across all treatment groups was deserved compared to the control (*p*< 0.0001). The halved reduction compared to the control was almost equal in TAS-115 and TAS-115 + DOXO groups, suggesting a potential link to the reduced MDA-MB-231 cell population in heterotypic spheroids. Compared to the DMSO group, c-MET levels were significantly lower in TAS-115 (*p* < 0.05) and TAS-115 + DOXO (*p* < 0.0001) groups, indicating that the effect of TAS-115 is independent of its solvent DMSO (figure 4(A)). Vascular endothelial growth factor (VEGF) plays a crucial role in tumor angiogenesis in breast cancer. Since TAS-115 inhibits c-MET/HGF and VEGF-mediated pathways, its impact on VEGF-*α* expression was also evaluated. The analysis showed a significant reduction in VEGF-*α* levels in all treatment groups compared to the control (*p*< 0.0001). With respect to the control, VEGF-*α* levels decreased to ∼34% in DMSO,∼43% in DOXO, ∼25% in TAS-115 and ∼22% in TAS-115 + DOX groups. Additionally, VEGF-*α* levels were significantly lower in TAS-115 (*p*< 0.001) and TAS-115 + DOXO (*p* < 0.0001) groups compared to the DMSO group, confirming the specific inhibitory effect of TAS-115 (figure 4(B)). TNF-*α* and IL-6 promote inflammation in the TME, facilitating breast cancer cell proliferation and metastasis. Analysis showed a significant reduction in TNF-*α* (figure 4(C)) and IL-6 (figure 4(D)) levels, normalized to the control group, across all treatment groups, with TAS-115 playing a major role in this decrease (*p*< 0.0001). Although TNF-*α* and IL-6 levels were lower in the TAS-115 + DOXO group compared to TAS-115 alone, the difference was not statistically significant. Additionally, TNF-*α* (figure 4(C)) and IL-6 (figure 4(d)) levels were significantly reduced in the TAS-115 and TAS-115 + DOXO groups compared to the DMSO group (*p*< 0.001). IL-1*α* is a potent pro-inflammatory cytokine secreted by immune and cancer cells. By inducing inflammation, it creates a favorable microenvironment for breast cancer cell growth and progression while also promoting tumor development through increased interactions between cancer and stromal cells [18]. The analysis showed that IL-1*α* levels normalized to the control group were significantly reduced in all treatment groups (*p* < 0.0001). Namely, this decrease was 64% in the TAS-115 group and 56% in the TAS-115 + DOXO group (figure 4(E)).

**Figure 5.**
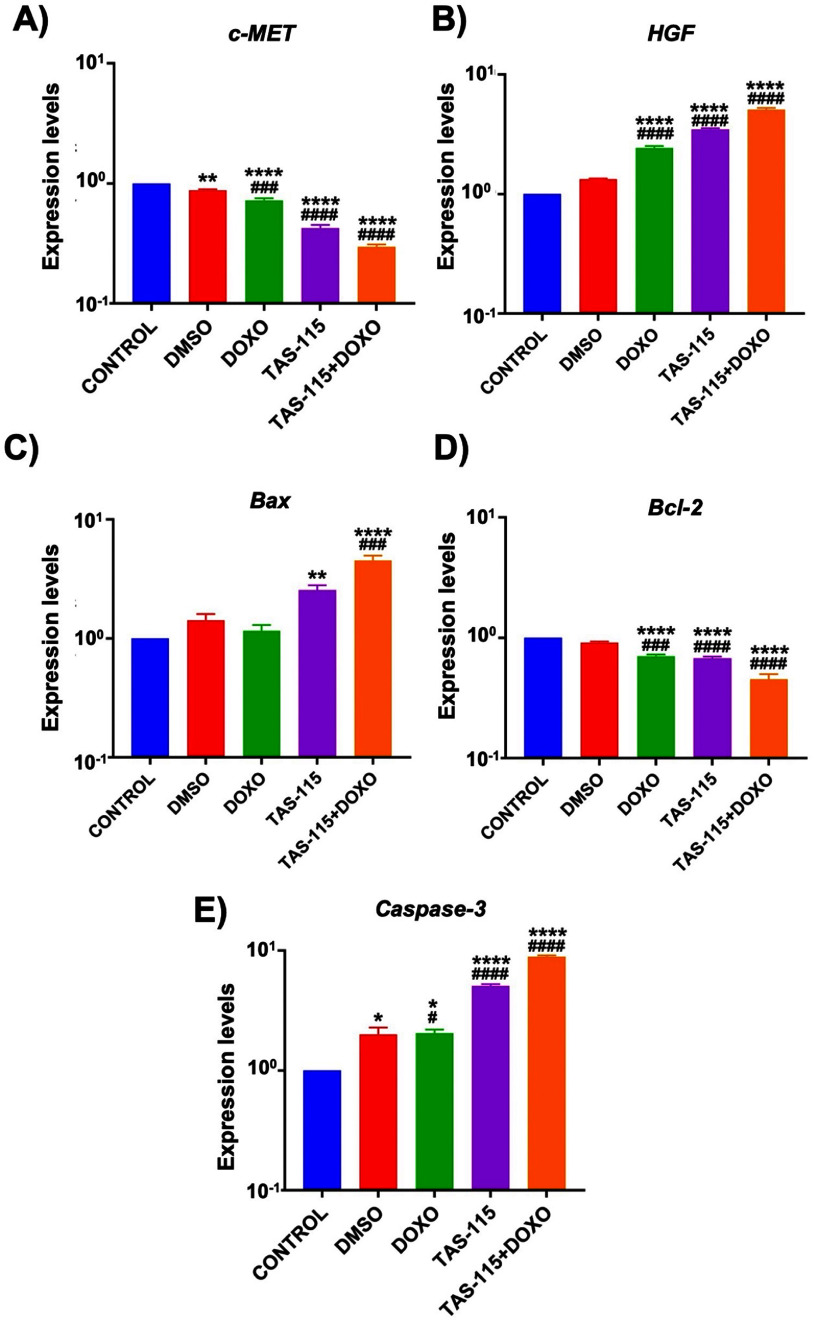
(A) *c-MET*, (B) *HGF*, (C) *Bax*, (D) *Bcl-2,* and (E) *Caspase-3* gene expression levels in heterotypic tumor spheroids following the TAS-115 treatment, both individually or in combination with DOXO, as assessed by qRT-PCR analysis (mean ± SD, *n*= 12) (p^*^ < 0.05, p^**^ < 0.01, p^***^ < 0.001, p^****^ < 0.0001; compared to control, *p*^#^ < 0.05, *p^##^*< 0.01, *p^###^* < 0.001, *p^####^* < 0.0001; compared to DMSO).

After performing a protein expression study, changes in *c-MET, HGF*, and *apoptosis-related Bax, Bcl-2*, and *Caspase-3* gene expression levels in spheroids following the treatment with TAS-115 and DOXO were evaluated using qRT-PCR. c-MET expression was significantly reduced in all treatment groups compared to both control and DMSO groups (*p*< 0.0001), consistent with immunofluorescence findings (figure 5(A)). Conversely, HGF expression was significantly elevated in the treatment groups relative to the control and DMSO groups (*p* < 0.0001), potentially related to fibroblast-derived HGF expression, suggesting a possible survival advantage (figure 5(B)). *Bax* gene expression, which is associated with apoptosis, increased by 2-fold (*p*< 0.01) in the TAS-115 group and 4-fold (*p* < 0.0001) in the TAS-115 + DOXO group compared to the control (figure 5(C)). On the other hand, *Bcl-2*, an anti-apoptotic gene, was markedly reduced in all treatment groups compared to both control and DMSO groups (*p* < 0.0001) with ∼ 32% reduction in the TAS-115 and ∼ 55% reduction in the TAS-115 + DOXO group (figure 5(D)). Finally, *caspase-3* gene expression, a critical executioner of apoptosis, was significantly elevated in all treatment groups compared to the control and DMSO groups (*p*< 0.0001), further supporting the observed changes in *Bax* and *Bcl-2* expression. Collectively, these findings demonstrated that TAS-115 effectively induced apoptosis (figure 5(E)).

### 3.4. Assessment of the effect of TAS-115 individual and co-administration with DOXO on the invasion potential of cancer cells from a heterotypic spheroid in a 3D bioprinted static model

To investigate the impact of TAS-115 treatment on cancer cell invasiveness, a 3D static model was employed in which heterotypic tumor spheroids were bioprinted at defined positions in the C2F3 composite hydrogel using aspiration-assisted bioprinting. Following overnight stabilization, the IC_50_ concentration of TAS-115, either alone or in combination with DOXO, was administered, and the tumor invasion was monitored over 24, 48, and 72 h.

**Figure 6.**
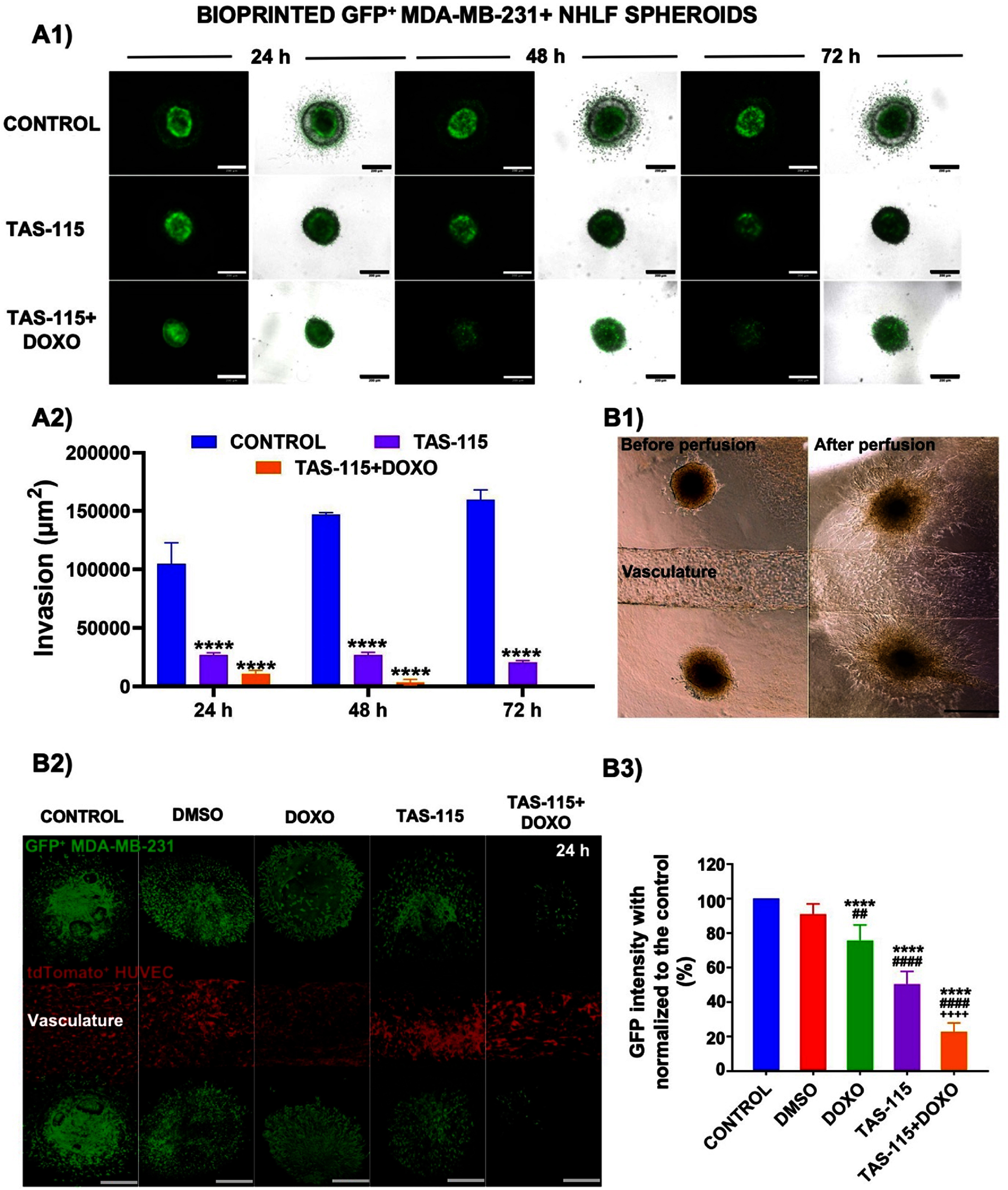
(A1) Multiphoton microscope images showing the effect of TAS-115 treatment, both individually or in combination with DOXO, on the ECM invasion of cancer cells from spheroids formed with GFP^+^ MDA-MB-231 cells and NHLF cells. The scale bar represents 200 *µ*m. (A2) A graph illustrating the degree of invasion (*n*= 5). (B1) Bright-field images of the control devices before and after 24 h of perfusion, showing that spheroid structure remained intact under flow. The scale bar represents 200 *µ*m. (B2) Multiphoton microscope images at 24 h post-perfusion depicting the effect of TAS-115, administered individually or in combination with DOXO, on the migration and metastatic potential of cancer cells after perfusion of devices. The scale bar represents 200 *µ*m. (B3) Quantitative analysis of GFP intensity revealed a significant decrease in the treatment groups compared to the control, where GFP intensity was considered to reflect cell viability rather than migratory capacity (p^*^ < 0.05, p^**^ < 0.01, p^***^ < 0.001, p^****^ < 0.0001; compared to the control, *p*^#^ < 0.05, *p^##^*< 0.01, *p^###^* < 0.001, *p^####^* < 0.0001; compared to DMSO) (mean ± SD, *n* = 5).

In the control group, cancer cells displayed a progressive invasion pattern, initiating as early as 24 h and markedly expanding their migratory fronts by 48 and 72 h, as evident from the significant enlargement of the invasive area surrounding the spheroids. In contrast, the treatment with TAS-115 alone markedly suppressed cancer cell dissemination, with spheroid structures remaining compact and exhibiting minimal peripheral cell migration throughout the observation period. Remarkably, the co-treatment group (TAS-115 + DOXO) exhibited the most pronounced anti-invasive effect. Although slight loosening was noted in spheroid compaction, no measurable invasion was detected by 72 h, indicating a complementary inhibitory effect of the co-treatment on both cellular migration and viability (figure 6(A1)). Quantitative image analysis revealed a statistically significant reduction in invasion areas in both TAS-115 and TAS-115 + DOXO groups compared to the untreated control (*p* < 0.0001) (figure 6(A2)). After 24 h, the degree of invasion was reduced by ∼4-folds in the TAS-115 and ∼ 10-folds in the TAS-115 + DOXO group compared to the control. After 48 h, the degree of invasion was decreased by∼ 40-folds, especially in the TAS-115 + DOXO group. Moreover, while TAS-115 alone exerted a notable anti-invasive effect, the addition of DOXO further enhanced this inhibition, completely abrogating cell invasion by 72 h. These findings underscore the potent anti-invasive capacity of TAS-115, particularly when used in combination with DOXO, in a more physiologically relevant 3D tumor model.

### 3.5. Assessment of the effect of TAS-115 individual and co-administration with DOXO on the migration potential of cancer cells from heterotypic spheroids in a 3D bioprinted perfusable model

The migratory and metastatic behavior of heterotypic spheroids was further investigated in a 3D bioprinted perfused model. Spheroids were strategically placed in proximity to a perfusion channel lined up with HUVECs, providing a dynamic microenvironment mimicking physiological perfusion.

In the perfusable 3D model, bright-field imaging of control constructs (figure 6(B1)) showed compact spheroids before perfusion maintained their structural integrity after 24 h of continuous perfusion. Fluorescence imaging after 24 h of perfusion (figure 6(B2)) revealed clear outward migration of GFP^+^ MDA-MB-231 cells toward and into the endothelialized channel in the control condition. Migrating cells displayed elongated morphologies consistent with mesenchymal movement, and infiltration into the vascular mimic was clearly observed in Z-stack reconstructions, particularly from the distal regions of the spheroid body. In contrast, the treatment with TAS-115 resulted in partial disruption of spheroid compaction, with fewer GFP^+^ cells detected outside the spheroid core. The migratory front was notably reduced, and cells appeared less polarized, suggesting inhibition of directed migration. Notably, the TAS-115 + DOXO group showed a more pronounced effect: spheroids were visibly smaller and disorganized, with a dramatic loss of GFP^+^ signal in the surrounding matrix. No significant invasion into the channel was observed, and GFP^+^ cells remained largely confined to the hydrogel, indicating a near-complete loss of metastatic behavior. Moreover, HUVECs lining the vascular channel appeared dense and confluent in the control group, supporting interactions with invading cancer cells. However, in both treatment groups, particularly in TAS-115 + DOXO, tdTomato^+^ endothelial signal was visibly sparser and disorganized, possibly indicating indirect effects of drug exposure through the perfused medium (figure 6(B2)). Quantitative analysis of GFP intensity revealed a significant decrease in the treatment groups compared to the control (*p* < 0.0001) (figure 6(B3)). The GFP signal intensity was ∼ 50% of the control in the TAS-115 group and ∼25% in the TAS-115 + DOXO group. The TAS-115 + DOXO was also significantly lower than TAS-115 alone (*p* < 0.0001) (figure 6(B3)). The DMSO condition displayed reduced outward movement in the micrographs and a modest decline in signal intensity relative to the control. Because GFP signal intensity predominantly reflects tumor-cell viability rather than migration distance, these measurements were reported as viability-linked readouts within the perfused system.

## 4. Discussion

TNBC is an aggressive subtype characterized by limited treatment options, high recurrence rates, and poor survival outcomes [20, 21]. Its progression is strongly influenced by the TME and signaling pathways such as the c-MET/HGF axis, which regulate proliferation, invasion, and metastasis [22, 23].

TAS-115, a multi-target tyrosine kinase inhibitor of c-MET, VEGFR, and PDGFR, has shown antitumor efficacy in several malignancies but has not yet been evaluated in breast cancer [24–27]. According to the available datasheet, TAS-115 (Pamufetinib) is insoluble in water and ethanol but soluble in DMSO, indicating a predominantly hydrophobic character. This hydrophobicity facilitates passive diffusion across the lipid bilayer of cancer cells, allowing TAS-115 to reach intracellular targets without requiring specific membrane transporters. Once inside the cell, TAS-115 inhibits receptor tyrosine kinases such as c-MET and VEGFR2, thereby disrupting pro-survival and proliferative signaling pathways. In this way, its hydrophobic nature supports efficient cell penetration and enhances its inhibitory activity on malignant cells. Despite the limited number of studies evaluating the efficacy of TAS-115 on various cancer types, no research has specifically investigated its effects on c-MET/HGF-mediated breast cancer progression and metastasis, either alone or in combination with DOXO. To fill this gap, our study aimed to develop a 3D heterotypic spheroid model. The model, designed to replicate the *in vivo* metastatic behavior of cancer cells to lungs, consists of TNBC GFP^+^ MDA-MB-231 cells and NHLFs. This combination aims to mimic the interaction between cancer cells and the lung microenvironment and provide insight into the metastatic process of TNBCs to the lungs. Since no previous studies have evaluated the administration of TAS-115 in breast cancer cells or reported an effective inhibitory dose for this compound, our study referenced effective dose ranges established for other cancer types [12, 24]. Using this framework, we assessed the effects of TAS-115, both individually or in combination with DOXO, on cell viability within heterotypic spheroids.

In a 2D *in vitro* study conducted by Yamada *et al* [9], TAS-115 demonstrated dose-dependent inhibition of proliferation in Yamato-SS (c-MET-dependent) and SYO-1 and HS-SY-II (PDGFR*α*-dependent) synovial sarcoma cell lines. Similarly, our study showed that TAS-115 significantly reduced cell viability and proliferation in GFP^+^ TNBC cells in a dose- and time-dependent manner, as assessed by Alamar Blue assays and Ki67 immunocytochemical staining. Notably, this reduction was more pronounced when TAS-115 was combined with DOXO, providing the first evidence of TAS-115’s impact on TNBC cell viability and proliferation. In line with findings from Yamada *et al* [9] and Kunii *et al* [12], our study showed that TAS-115 inhibited c-MET phosphorylation and the activation of downstream signaling molecules, such as Akt and ERK1/2. Immunohistochemical analysis of 3D heterotypic tumor spheroids confirmed that both individual and combined treatment with TAS-115 and DOXO reduced the expression of c-MET, p–c-MET, and p-mTOR, a key node in the PI3K/Akt/mTOR pathway.

In this study, expression levels of key apoptotic and survival-related genes, including *Bax, Bcl-2, Caspase-3, c-MET*, and *HGF* in response to TAS-115 and its combination with DOXO in 3D tumor spheroids. The results provide valuable insights into the molecular mechanisms underlying the effects of TAS-115 on breast cancer progression, particularly in terms of apoptosis, cell survival, and the c-MET/HGF signaling axis. Our data revealed a significant modulation of *Bax* and *Bcl-2* expression in response to the TAS-115 and DOXO treatment. *Bax*, a pro-apoptotic gene, was significantly upregulated in treated spheroids, indicating an induction of apoptotic signaling pathways. This upregulation of *Bax* was particularly pronounced in the combinatorial treatment group, suggesting a complementary effect of TAS-115 and DOXO in promoting apoptosis in TNBC cells. On the other hand, *Bcl-2*, an anti-apoptotic protein, was downregulated in the treated groups, supporting the notion that TAS-115 disrupts the balance between pro- and anti-apoptotic signals, favoring cell death. These findings align with previous studies that have demonstrated the ability of tyrosine kinase inhibitors, such as TAS-115, to modulate apoptotic pathways and sensitize cancer cells to chemotherapy [9, 24]. Although a phase I clinical trial evaluating TAS-115 in various solid tumors observed tumor size reduction in some cases [10], the study involved only a single breast cancer patient, limiting the ability to draw conclusions about TAS-115’s effectiveness in breast cancer. Nevertheless, the decrease in tumor tissue observed in our 3D model mirrors the loss of compaction in cancer cells after the TAS-115 treatment, consistent with *in vivo* tumor regression effects.

In this study, we demonstrated that TAS-115 effectively induces apoptosis in heterotypic tumor spheroids, and that its combination with DOXO further amplifies this effect. The significant increase in Caspase-3/7 activity, particularly in the TAS-115 + DOXO group, indicates a complementary interaction, enhancing apoptotic signaling beyond the levels achieved by either agent alone. Caspase-3 and −7 are key executioners of the apoptotic cascade, and their robust activation confirms the pro-apoptotic efficacy of TAS-115 in this 3D tumor model, which more accurately reflects the *in vivo* TME compared to the conventional 2D culture [28]. The synergistic the interaction between TAS-115 and DOXO likely arises from their distinct yet complementary mechanisms of action converging at the level of cancer cell survival. TAS-115, as a multi-targeted kinase inhibitor, disrupts pro-survival signaling by blocking c-MET/HGF and VEGFR2 pathways, thereby attenuating downstream PI3K/Akt/mTOR activation and diminishing the ability of cancer cells to withstand stress [29]. Conversely, DOXO exerts its cytotoxicity through topoisomerase II inhibition, inducing DNA double-strand breaks and triggering mitochondrial apoptotic cascades. Within this context, TAS-115 effectively weakens oncogenic signaling and resistance networks, creating a permissive state that enhances the susceptibility of tumor cells to DOXO-induced DNA damage. Consequently, while TAS-115 alone significantly impairs tumor growth, its co-administration with DOXO amplifies apoptotic signaling and further suppresses tumor cell survival, underscoring the therapeutic promise of this combinatorial strategy in TNBC [30]. In addition to its apoptotic effects, our findings reveal a previously unreported anti-inflammatory property of TAS-115. Specifically, TAS-115 alone and in combination with DOXO significantly reduced the levels of key pro-inflammatory cytokines, including TNF-*α*, IL-1*α*, and IL-6, which are often elevated in the TME and contribute to tumor progression, therapy resistance, and immunosuppression. The ability of TAS-115 to suppress these cytokines suggests that its therapeutic efficacy may extend beyond direct tumor cell killing to modulation of the tumor immune milieu [31]. To our knowledge, this is the first study to investigate the impact of TAS-115 on cytokine secretion in a 3D tumor context, providing novel insights into its potential anti-inflammatory and immunomodulatory actions.

In our study, the primary focus was to evaluate the impact of TAS-115 on c-MET-driven proliferation and migration. While we did not directly assess tumor repolarization, it is reasonable to propose that inhibition of the c-MET/HGF axis may indirectly promote a shift in the TME. Specifically, c-MET signaling is known to support an immunosuppressive and pro-angiogenic milieu through the induction of cytokines such as IL-6 and TNF-*α*. By attenuating these pathways, TAS-115 could potentially repolarize the TME from a pro-tumorigenic state toward a more anti-tumorigenic phenotype. However, this effect was not directly measured in the current study and remains an interesting avenue for future investigations.

Interestingly, while TAS-115 reduced the survival of tumor cells, it appeared to support NHLF viability, as indicated by HGF expression. This differential response may reflect the unique role of the stromal fibroblasts in the TME. Fibroblasts have been shown to play a supportive role in tumor growth and metastasis, often through the secretion of growth factors and cytokines [32]. It is possible that TAS-115, by inhibiting c-MET and other receptor tyrosine kinases, may alter the signaling interactions between cancer cells and stromal cells, leading to the enhanced survival of NHLFs. This finding suggests that while TAS-115 is effective in targeting tumor cells, its effects on stromal cells may need to be carefully considered when designing combinatorial therapies. The observed increase in HGF expression, which may indicate a relative survival advantage of NHLFs, raises important questions about the TME and the complex interplay between cancer cells and stromal components. Previous studies have suggested that therapies targeting cancer cells alone may not be sufficient, as stromal cells can contribute to drug resistance and tumor progression [33, 34]. Therefore, the dual effects of TAS-115 on both tumor cells and stromal cells may offer new insights into the development of more effective therapeutic strategies for TNBC, where the TME is a critical factor in treatment outcomes.

In this study, we demonstrated that TAS-115, a multi-targeted tyrosine kinase inhibitor, effectively suppresses the invasive and migratory potential of breast cancer cells in heterotypic spheroids in a hydrogel matrix. While cancer cells in the control group exhibited progressive invasion from the spheroid core over time, the TAS-115 treatment-maintained spheroid compaction and significantly reduced cellular outgrowth. These anti-invasive effects were even more pronounced when TAS-115 was co-administered with DOXO, as invasive cells were nearly absent at 72 h. Furthermore, in the 3D perfusion model, GFP^+^ MDA-MB-231 cells displayed a reduced migratory pattern and diminished viability under TAS-115 and TAS-115 + DOXO treatments, with the most substantial suppression observed in the combinatorial group. Previous studies have demonstrated that activation of the HGF/c-MET pathway promotes invasive behavior, angiogenesis, and metastasis in TNBC models, particularly in MDA-MB-231 and BT549 cell lines [35]. Our data support and expand upon these findings by showing that TAS-115 can counteract these effects within a physiologically relevant 3D microenvironment composed of cancer cells, fibroblasts, and endothelial cells embedded in a perfusable matrix. The decrease in both GFP^+^ cancer cells and tdTomato^+^ endothelial cells within the perfusable channels suggests that TAS-115 not only disrupts cancer cell invasion and proliferation but also impairs tumor-associated angiogenesis. This dual inhibition of tumor–stroma and tumor-vascular interactions provides a more comprehensive suppression of the metastatic cascade [36]. By leveraging a complex *ex vivo* system that better recapitulates the *in vivo* TME, we provide compelling evidence for the potential of TAS-115 as a therapeutic agent in limiting metastatic spread, especially when used in combination with DOXO. The methodological distinction between the invasion and migration assays was intentional, reflecting their unique mechanistic focuses. The invasion analysis, performed under static conditions, allowed us to isolate the effects of matrix remodeling and cellular protrusion into the hydrogel. In contrast, the migration model incorporated a perfused vascular channel, enabling assessment of spheroid-directed movement under flow and endothelial influence, thereby better mimicking *in vivo* microenvironmental dynamics. In this context, although GFP intensity primarily reflects cancer cell viability, in the perfusable migration model it also paralleled the reduced outward movement of GFP^+^ cells from spheroids toward the vascular conduit. This decline was more pronounced under perfused conditions, likely due to the continuous exposure of spheroids to the compounds, resulting in more efficient drug penetration. These considerations highlight the importance of model-specific factors when interpreting drug responses and indicate that complementary readouts, such as spheroid expansion or direct tracking of migrating cells, may further refine the assessment of migratory capacity.

In summary, our findings demonstrate that TAS-115, either alone or in combination with DOXO, significantly reduces tumor cell survival, accompanied by downregulation of key apoptotic and survival-related genes. Notably, an observed increase in HGF expression may reflect a relative survival advantage of NHLFs, underscoring the need for further investigation into stromal responses to TAS-115. Mechanistically, DOXO induces DNA damage and apoptosis via topoisomerase II inhibition, whereas TAS-115 primarily blocks pro-survival signaling through c-MET/HGF and VEGFR2 pathways. These distinct yet complementary actions provide a rationale for considering TAS-115 not only as a promising stand-alone therapeutic option but also as a potential adjunct to chemotherapy in TNBC.

## 5. Conclusion

This study demonstrates the robust antitumor efficacy of TAS-115, both as a monotherapy and in combination with DOXO, in 3D heterotypic breast cancer models that recapitulate critical features of the TME. TAS-115 markedly inhibited cancer cell proliferation, as shown by reduced GFP^+^ MDA-MB-231 cell populations, and induced apoptosis via upregulation of *Bax* and *Caspase-3*, alongside suppression of *Bcl-2* expression. Mechanistically, TAS-115 downregulated c-MET and its downstream mTOR signaling, indicating a targeted disruption of survival and growth pathways in metastatic breast cancer cells. The combined treatment with DOXO further potentiated these effects, promoting enhanced apoptotic activity and suppressing the migratory and invasive capacities of tumor cells. Moreover, TAS-115 significantly attenuated tumor-promoting cytokines, including TNF-*α*, IL-6, and VEGF-*α*, suggesting a potential role in modulating the inflammatory TME. Interestingly, while exerting cytotoxic effects on cancer cells, TAS-115 was associated with an increase in HGF expression, which may indicate a relative survival advantage of NHLFs. This suggests a potential differential impact on stromal components, which may have implications for tumor–stroma interactions and therapy design. Collectively, these findings underscore the therapeutic promise of TAS-115, particularly in combination with conventional chemotherapeutics, as a multi-faceted agent capable of suppressing tumor growth, metastasis, and inflammation. Given the complex interplay between cancer cells and their microenvironment, integrating TAS-115 into combinatorial treatment regimens may represent a promising strategy for overcoming therapy resistance and improving clinical outcomes in TNBC.

## Data Availability

The data cannot be made publicly available upon publication because they are not available in a format that is sufficiently accessible or reusable by other researchers. The data that support the findings of this study are available upon reasonable request from the authors.
